# Bone formation transcripts dominate the differential gene expression profile in an equine osteoporotic condition associated with pulmonary silicosis

**DOI:** 10.1371/journal.pone.0197459

**Published:** 2018-06-01

**Authors:** Regina Zavodovskaya, Susan M. Stover, Brian G. Murphy, Scott Katzman, Blythe Durbin-Johnson, Monica Britton, Carrie J. Finno

**Affiliations:** 1 Department of Anatomy, Physiology and Cell Biology, UC Davis School of Veterinary Medicine, University of California, Davis, California, United States of America; 2 Department of Pathology, Microbiology and Immunology, UC Davis School of Veterinary Medicine, University of California, Davis, California, United States of America; 3 Department of Surgical & Radiological Sciences, UC Davis School of Veterinary Medicine, University of California, Davis, California, United States of America; 4 Department of Public Health Sciences, UC Davis School of Medicine, University of California, Davis, Davis, California, United States of America; 5 UC Davis Genome Center, Bioinformatics Core Facility, University of California, Davis, Davis, California, United States of America; 6 Department of Population Health & Reproduction, UC Davis School of Veterinary Medicine, University of California, Davis, California, United States of America; Augusta University, UNITED STATES

## Abstract

Osteoporosis has been associated with pulmonary silicosis in California horses exposed to soils rich in cytotoxic silica dioxide crystals, a syndrome termed silicate associated osteoporosis (SAO). The causal mechanism for the development of osteoporosis is unknown. Osteoporotic lesions are primarily located in bone marrow-rich sites such as ribs, scapula and pelvis. Gene transcription patterns within bone marrow and pulmonary lymph nodes of affected horses may offer clues to disease pathobiology. Bone marrow core and tracheobronchial lymph node tissue samples harvested postmortem from affected and unaffected horses were examined histologically and subjected to RNA sequencing (RNA-seq). Sequenced data were analyzed for differential gene expression and gene ontology. Metatranscriptomic and metagenomic assays evaluated samples for infectious agents. Thirteen of 17 differentially expressed transcripts in bone marrow were linked to bone and cartilage formation such as integrin binding bone sialoprotein (log_2_FC = 3.39, P_FDR_ = 0.013) and chondroadherin (log_2_FC = 4.48, P_FDR_ = 0.031). *Equus caballus* solute carrier family 9, subfamily A2 (log_2_FC = 3.77, P_FDR_ = 0.0034) was one of the four differentially expressed transcripts linked to osteoclast activity. Osteoblasts were hyperplastic and hypertrophic in bone marrow from affected horses. Biological pathways associated with skeletal morphogenesis were significantly enriched in affected horses. The 30 differentially expressed genes in affected lymph nodes were associated with inflammatory responses. Evidence of infectious agents was not found. The SAO affected bone marrow molecular signature demonstrated increased transcription and heightened activation of osteoblasts. Increased osteoblastic activity could be part of the pathological mechanism for osteoporosis or a compensatory response to the accelerated osteolysis. Transcriptome data offer gene targets for inquiries into the role of osteocytes and osteoblasts in SAO pathogenesis. Viral or bacterial infectious etiology in SAO is less likely based on metatranscriptomic and metagenomic data but cannot be completely ruled out.

## Introduction

Progressive osteoporosis is a comorbid condition in horses that have chronic pulmonary silicosis.[1] Clinical manifestations of osteoporosis include debilitating skeletal deformities, cervical osteoarthritis, and pathological fractures.[1, 2] Lytic bone lesions are typically found in ribs, vertebrae, scapulae, and the pelvis.[1, 3] Osteoporosis is more strongly associated with silicotic lymphadenitis in the nodes that drain the lung parenchyma (tracheobronchial lymph nodes (tLNs)), than with pulmonary silicosis.[1] The condition is termed silicate associated osteoporosis (SAO) when the skeletal and thoracic (lung and tLN) lesions are present simultaneously.[1] Cases of SAO are geographically clustered in regions with soil containing high levels of cytotoxic silicates (e.g., cristobalite—SiO_2_ polymorph), which are aerosolized and inhaled as fine crystalline silicate particles. [2, 4] Other causes of chronic granulomatous pulmonary disease have not been found in affected horses.[1] The pathogenic link between silicosis and osteoporosis remains putative.

Abnormal bone remodeling and anomalous osteoclast morphology (hypertrophy, hyperplasia), coupled with excessive osteolysis in SAO, are histologically similar to the lytic phase of Paget’s disease of bone (PDB) in people.[5] However, the chronic pulmonary inflammation induced by cytotoxic silicates, age of onset, skeletal distribution, and progressive osteolysis set SAO apart from PDB [1, 3, 5] (reviewed by Nebot Valenzuela et al. [6]). The roles of immunological background and other contributing factors for the development of SAO, such as dose and duration of exposure to the silicate crystals warrant further investigation. A previous investigation did not reveal viral or bacterial infections as cofactors in SAO through culture and microscopic examination of affected tissues, however broad metatranscriptomic and metagenomic assays to look for novel infectious agents have not been performed.[1] Though bone lesions have been well characterized grossly and morphologically in SAO, the underlying cause of the pathologic osteolysis is unknown.

SAO has only been reported in horses. However, singular cases of similar comorbidities have been suspected to occur in humans. [7–9] Osteoporosis has been associated with pulmonary diseases such as cystic fibrosis [10] and chronic obstructive pulmonary disease in people.[11, 12] Other extra-skeletal inflammatory diseases associated with osteoporosis include inflammatory bowel disease [13] and systemic lupus erythematosus. [14] Hyper-activation of osteoclasts during systemic inflammation suggests that a subset of pro-inflammatory signals may induce osteoporosis.[15]

Merging of osteoclast activation and pro-inflammatory signals is in part supported by molecular studies. Osteoclast differentiation and activation are regulated mainly through an interaction between “receptor activator of nuclear factor κ-β” (RANK) and its ligand (RANKL).[16] Pro-inflammatory cytokines like IL1, TNFα and IL6 potentiate RANK downstream signaling cascades, or increase paracrine production of RANKL. Thus, these pro-inflammatory cytokines can increase osteoclast activity.[17] Therefore, osteolysis in SAO may be derived from chronic cytotoxic silicate-induced inflammation within the respiratory tract. We hypothesized that the inflammatory trigger initiates aberrant osteoclastogenesis in the bone marrow (BM), where osteoclast precursors receive systemic signals, resulting in accelerated osteolysis and osteoporosis in affected horses.

To test our hypothesis we generated differential gene expression (DGE) profiles of the main tissue sites affected by SAO, the rib BM and tLN, in affected and unaffected horses. Because of the hypothesized systemic effect of pro-osteoclastogenic trigger, BM samples were collected away from gross lesions of osteoporosis with markedly altered cell populations. This tissue collection protocol was designed to capture the pattern of cellular signals before significant tissue heterogeneity would dominate the transcriptional differences. The presence of transcripts from infectious agents in the target tissues was also evaluated.

## Material and methods

### Animals

Samples from the two tissue targets, BM cores and tLNs, were collected post mortem from sex and age-matched SAO^+^ and SAO^-^ horses (S1 Table). There is no known breed predisposition for SAO.[1] To prevent inclusion of subclinically affected horses in the control group, inclusion criteria for control horses included absence of exposure to SAO endemic regions (i.e., exposure to cytotoxic silica dioxide particles) and absence of granulomatous pulmonary disease on post mortem examination. Study animals consisted of 8 SAO-affected, (3 females: Appaloosa, Quarter Horse and Thoroughbred, and 5 males: Holsteiner, Quarter Horse cross, Quarter Horse, Paint and Arabian), and 8 unaffected horses, (4 females: Hanoverian, Thoroughbred, Paint and Quarter Horse, and 4 males: Quarter Horse, Standardbred/Arabian cross, Mustang cross, and Morgan/Arabian cross). The median age of SAO^+^ horses in BM tissue group (n = 8, 20 years, range 12 to 30) was not significantly different (Mann-Whitney test, p = 0.46) from the median age of unaffected horses (n = 8, 17.5 years, range 8 to 26). Similarly, the median age of SAO^+^ horses in tLN tissue group (n = 7, 19 years, range 12 to 30) was not significantly different (Mann-Whitney test, p = 0.48) from the median age of the unaffected horses (n = 6, 17 years, range 11 to 26). Clinical disease in SAO horses was categorized as mild or severe based on the absence or presence, respectively, of gross skeletal deformities. Formal consent was obtained from owners for horses donated to the study. All horses were euthanized with an intravenous overdose of pentobarbital sodium (*FATAL-PLUS—*Pentobarbital Sodium 390 mg/mL, Vortech Pharmaceuticals, Dearborn, MI) solution (>100 mg/kg) due to poor quality of life (SAO horses) or neurologic, chronic focal musculoskeletal or behavioral abnormalities, or trauma (S1 Table). The project was approved by the University of California Davis Institutional Animal Care and Use Committee.

### SAO clinical phenotyping

The medical history included known exposure to SAO endemic regions. Musculoskeletal conditions including history of skeletal deformities, lameness, fracture(s), decreased range of motion, and exercise intolerance, as well as abnormalities of the respiratory system were investigated and documented. Ante mortem clinical evaluation included physical examination and complete blood cell count and serum biochemistry including ionized calcium concentrations. All horses in the study received non-steroidal anti-inflammatory (NSAIDs) medications (e.g. flunixin meglumine, firocoxib, or phenylbutazone) at standard dosing regiments for pain management prior to euthanasia. Horses treated with bisphosphonates, including zoledronic acid (Zometa), clodronate (Osphos) and tiludronate (Sigma), within the past twelve months were excluded from the study. The diagnosis of SAO was confirmed by post mortem evidence of silicosis in the tLN and osteoporosis.[1]

### Post mortem evaluation

The skull, scapulae, humeri, ribs, pelvis, lungs, lymph nodes (cervical, thoracic and abdominal), endocrine organs (pituitary, thyroid, and adrenal glands; parathyroid glands were examined in 3 of the affected and 2 control horses due to the difficulty in finding the parathyroid glands in this specie), skeletal muscles, major abdominal organs and reproductive organs (when present) were evaluated grossly and histologically. Sections of the 7^th^, 8^th^, 9^th^, or 10^th^ rib and other bones were evaluated microscopically to confirm SAO status when bone deformity, porosity, fracture, or discoloration were present.

### Histology

All tissues were fixed in 10% buffered formalin. Rib BM cores specimens were decalcified with 10% ethylenediaminetetraacetic acid (EDTA; Amresco, Solon, OH) at 4°C for 2 weeks prior to trimming and sectioning. Trimmed tissues underwent routine histological processing resulting in 4μm sections stained with hematoxylin and eosin using routine protocols.

Microscopic features used to confirm silicosis in tLNs included necrotizing and fibrosing granulomas with intralesional refractile crystals. Features used to confirm osteoporosis included atypically large and numerous intralesional osteoclasts with increased numbers of nuclei, and abnormal bone tissue remodeling characterized by haphazard, anastomosing, thin, woven and lamellar bone trabeculae with mosaic cement lines in areas of prior lysis.

Bone formation and resorption ratios were calculated using the length (in μm) of bone surface covered by osteoblasts or scalloped sites of resorption (Howship's lacunae), respectively, to the total length of bone surface (bone perimeter) using digital images of histological sections obtained at 100x magnification (image area = 877x666μm). Image software (Adobe Photoshop CS6, Adobe Systems Incorporated, San Jose, CA) was used for the analysis of images with sufficient morphological detail for cell and tissue identification (2–6 per specimen). The measuring scale was set to the calibration bar embedded in the images. Bone formation surface was defined as bone surface length in μm lined by osteoblasts. Bone resorption surfaces were defined by length in μm of scalloped bone surface contours. Osteoblast height, as a reflection of osteoblast hypertrophy and activity, was defined as the average apical to basilar distance in μm of 3 to 5 osteoblasts per image. Bone surfaces with artifacts and/or indistinct cellular morphology as well as surfaces created at the image edges were excluded from analysis. The calculated ratios were compared between SAO^+^ and unaffected horses using a two-sided t-test with p<0.05 considered statistically significant.

### Tissue targets for transcriptome sequencing and DGE analysis

Rib BM cores and tLN are characteristically affected in SAO and, therefore, were the target sites for *in situ* transcription profiling of SAO^+^ and unaffected horses. Rib BM core samples were collected away from grossly visible rib lesions.

The interval from euthanasia to the time of BM collection ranged from 20 min to 2 hours. However, one sample (SAO6) was preserved at 4°C for 12hrs post mortem and included in the study after passing RNA quality assessment (defined in the next paragraph). BM was collected aseptically by removing a segment of cortical bone with handsaw (Bosch PS50-2B, Mt. Prospect, IL) at the middle of the rib (7, 8, 9 or 10). The BM sample included both trabecular bone and inter-trabecular tissues. Transverse segments of tLN, including the cortex and medulla, were collected during necropsy, within 1–4 hours after euthanasia. Approximately 5 mm cubes of BM and tLN samples were placed into individual 1.8 ml cryogenic tubes (Nunc CryoTubes Sigma-Aldrich, Roskilde, Denmark) filled with 1.5mL of RNA–preserving solution (RNA*later*, Vilnius, Lithuania) and immediately cryopreserved in liquid nitrogen. Samples were stored at -80°C until RNA extraction. Next, a second set of samples of the BM and tLNs from the same site were fixed in 10% buffered formalin for microscopic examination.

### RNA extraction and quality validation

The tissues stored in RNA*later* were thawed on ice and sectioned using sterile instruments into ~0.3 cm cubes. The tissue cubes were individually crushed within liquid nitrogen to a fine powder using a sterile and UV-light-treated ceramic mortar and pestle also cleaned with RNase inhibiting solution (RNaseZAP wipes, Sigma-Aldrich, Saint Louis, MO). Total RNA was extracted using a commercial kit (RNeasy Plus Mini Kit, Qiagen, Hilden, Germany) following manufacturer’s protocol. Any contaminating genomic DNA was degraded enzymatically (RNase-Free DNase I and RNeasy Mini Elute Clean Up kits, Qiagen). An aliquot of the resulting total RNA with absorbency ratio (A_260nm/280nm_) of >1.9 was quantified spectrophotometrically (NanoDrop 1000, ThermoFisher Scientific, Waltham, MA) at 260nm wavelength. The quality of the RNA was assessed using a capillary electrophoresis technique (Agilent 2100 Bioanalyzer, Agilent Technologies, Santa Clara, CA, (S1 Table)).

### RNA-seq library construction and RNA sequencing

Samples of total RNA (500ng) with RIN of ≥ 7.5 were submitted to the DNA Technologies Core (University of California Davis Genome Center) and used for construction of strand-specific, poly-A enriched libraries. Following stranded mRNA-Seq Kit (KAPA Stranded mRNA-Seq Kit, Illumina platform, Cape Town, South Africa) manufacturer’s protocol, poly-A enrichment followed by cDNA synthesis and A tailing adapter ligation was performed. Library amplification was completed in 10 PCR cycles. Capillary electrophoresis technique (Agilent 2100 Bioanalyzer, Agilent Technologies, Santa Clara, CA) assessed distribution of the library fragments. Libraries were pooled and sequenced across two lanes with specification of 100-bp paired-end reads (100PE), (Illumina HiSeq 3000, San Diego, CA).

### Sequence reads quality assessment, trimming, and alignment

Raw reads were processed with expHTS [18] to trim low quality reads and adapter contamination, and to remove PCR duplicates. Trimmed reads for each sample were aligned to the EquCab2 horse genome with EquCab2.83 annotation (Ensembl), using STAR v. 2.5.1a aligner, [19] which also generated raw read counts per gene that were the input for the statistical analysis.

### Differential gene expression analyses

Differential gene expression analyses were conducted using the statistical limma-voom pipeline (version 3.28.17) [20] [21] and ANOVA statistical model that factors in presence of disease (affected/ unaffected), osteoporosis phenotype (mild, severe), tissue type (BM, tLN), and RNA isolation batch. Statistical significance of the genes was associated with an adjusted p value of <0.05. GO enrichment analysis was conducted (Bioconductor package topGO: Enrichment Analysis for Gene Ontology. R package version 2.24.0) to identify biological functions enriched in SAO^+^ tissues transcriptome.[22]

### PANTHER pathway analysis

The files of genes from the DGE analysis with ENSEMBL gene IDs and fold-change expressed in log_2_ for BM and LN tissues were loaded into Gene List Analysis (http://www.pantherdb.org, https://doi.org/10.1093/nar/gkw1138). The settings included statistical enrichment test defaults and *Equus Caballus* (EquCab2.0) as the reference species. Statistical significance was set as p-value below the Bonferroni correction was used to accounted for multiple comparisons in the analysis. PANTHER Pathways and GO-Slim Biological Processes were selected as the Annotation Data Sets for analysis.

### BRAKEN (Bayesian Re-estimation of Abundance after Classification with KrakEN) analysis

Because the pathogenesis of SAO is unknown, non-equine reads were evaluated for similarity to sequences of infectious agents that could be attributed to viruses or bacteria. Trimmed reads were evaluated with KrakEN, a k-mer based metagenomic classification tool, using a combined horse, viral, and bacterial database, followed by the BRAKEN metagenomic classification pipeline for transcription frequency of non-equine genetic material, and reported as fractions relative to the *Equus Caballus* taxonomy labels assigned by KrakEN.[23–25]

### Metagenomic analysis, viral discovery

Metagenomic analysis evaluated SAO^+^ tissues for viral transcripts. Samples of SAO^+^ BM and tLN SAO1, SAO7, SAO3, SAO9 (not used in transcriptome study)), as well as buffy coat (SAO7, SAO9) and lung (SAO1, SAO3) tissues were pooled. Viral capsid-protected nucleic acid was extracted and enriched according to an established protocol.[26] An Illumina MiSeq library was constructed using random RT-PCR followed by use of the Illumina Nextera kit and sequenced on an Ilumina MiSeq platform using 250 bases paired end. Sequence reads were de novo assembled using the Ensemble program and contigs and singletons then translated into hypothetical protein sequences, which were compared to all viral proteins in the NCBI virus RefSeq database using BLASTx. Detailed descriptions of the methods used in this analysis are included as supplemental material (S1 Protocol).

### RNA mapping statistics

Sufficient depth of sequencing and an adequate mapping rate were achieved for the DGE analysis. The average reads across BM and tLN samples were ~25.7 million reads/sample. Following trimming adapter contamination and removal of low quality reads and PCR duplicates with expHTS software, the average mapping rate to EquCab2.0 was 92.63%, where 54.6% reads uniquely aligned to an annotated horse gene and 32.4% aligned to regions of genome without annotation. 5.28% of reads aligned to multiple regions in the genome and 0.35% aligned with some annotation overlap, therefore, these reads were not assigned to a specific gene.

### Samples segregate based on tissue type and disease phenotype

The biologically relevant segregation of the sampled tissues in the MDS plots demonstrated high quality of starting genetic material, sequencing, and mapping techniques (S1–S3 Figs). The MDS plots illustrated that the clustering of SAO^+^ and SAO^-^ transcriptomes in BM and tLN tissues were most influenced by the *tissue origin* (S1 Fig), and the *disease phenotype* (S2 and S3 Figs), rather than biological variations such as breed or age (S5 and S6 Figs) among horses. SAO^+^ BM samples with mild osteoporosis phenotype clustered with unaffected samples (S2 Fig).

## Results

### Differential gene expression in BM and tLN

The ANOVA statistical model that accounted for the osteoporosis phenotype resulted in 17 significantly differentially expressed genes in BM and 36 significantly differentially expressed genes in tLN tissue (list of selected genes in Tables 1 and 2 and S2 and S3 Tables).

**Table 1:** Seventeen differentially expressed genes in SAO^+^ BM tissue listed in tissue function groups with base 2 logarithmic fold change and false discovery adjusted p-value.

Tissue Function	Gene	Specific Function	Log_2_FC, (P_FDR_)
Bone resorption	Equus caballus solute carrier family 9, subfamily A2 (*SLC9A2*)	Na^+^/H^+^ exchanger, cation proton antiporter; expressed in osteoclasts [27]	3.77 (0.034)
Bone resorption, formation, and maintenance	Osteomodulin, Osteoadherin (*OMD*)	Bone homeostasis and mineralization couples activity of Oc and Ob [28, 29]	3.92 (0.013)
	Distal-less homeobox3 (*DLX3*)	Homeobox gene embryonic bone development, Ob differentiation and Os function; Oc differentiation [30, 31] Higher expression in Os compared to Ob [32]	4.49 (0.033)
	*Transient receptor potential cation channel, subfamily V, member 4 (*TRPV4*)	Skeletal development at the growth plate; cartilage homeostasis [33–35] and osteoclast differentiation [36]	3.77 (0.030)
Bone homeostasis and formation	Collagen type XXIV, alpha 1 (*COL24A1*)	Precedes collagen I deposition in ossification centers [37]	4.18 (0.013)
	Sp7 transcription factor (*SP7*), Osterix (Osx)	Ob and chondrocyte differentiation from mesenchyme [38–40]; functions downstream of Runx2, BMP, Wnt [41]	3.32 (0.031)
	Integrin binding sialoprotein, bone sialoprotein (*BSP*)	Mineralization of bone and cartilage during bone development [42]	3.93 (0.013)
	Osteocalcin; bone gamma-carboxyglutamate (gla) protein (*BGLAP*)	Bone mineralization; regulated by Runx2 and Dlx3 and produced by in BM pluripotent cells during osteogenesis [31, 43, 44]	2.44 (0.029)
	Sphingomyelin phosphodiesterase 3 (*SMPD3*)	Endochondral bone formation in skeletal development [45, 46]	2.70 (0.021)
	Collagen type XI, alpha 2 (*COLXIA2*)	Skeletal development as cartilage ECM, embryonic development of the skeleton [47]	3.37 (0.029)
	Chondroadherin (*CHAD*)	Embryonic development of the skeleton, [48] minor component of bone matrix, [49] higher expression in Ob than Os [32]	4.48 (0.031)
	Anoctamin 5 (*ANO5*)	Embryonic development developing somites; Growth-plate chorndrocytes; Ob [50]	3.94 (0.037)
	Hydroxysteroid (17-beta) dehydrogenase 6 (*HSD17B6*)	Epimerization / interconversion estrone (E1) and estradiol (E2) [51]	-2.99 (0.013)
	WAP four-disulfide core domain 1 (WFDC1)	Secreted protease inhibitor with higher expression in Ob than Os [32]	2.67 (0.038)
	Cadherin 15 (CDH15)	Cell adhesion molecule with higher expression in Os than Ob [32]	3.97 (0.034)
Inflammation	Death-associated protein kinase 2 (*DAPK2*)	Granulocytes migration with activation and differentiation [52]	2.36 (0.031)

*Genes with functional overlap in Ob (osteoblast), Os (osteocyte) and Oc (osteoclast)

**Table 2:** Differentially expressed genes in tLN and base 2 logarithmic fold change with false discovery adjusted p-value. The selected three of the 36 differentially expressed genes are listed for their relevance to SAO.

Gene (Names and symbol)	Function	Log_2_FC, (P_FDR_)
Pentraxin 3 (*PTX3*)	Macrophages, dendritic cells and stromal cells soluble pattern recognition receptor (response to insults like silicates, infection and trauma);[53] identified as a promoter in TNF-α and IL-1β production through binding NF-κB elements *in vitro* [54]	3.9 (0.029)
Equus caballus Fc fragment of IgA receptor (*FCAR*)	Mediates immunological response in myeloid cells including alveolar macrophages that interact with IgA engaged with its antigen [55]	2.20 (0.030)
Dentin matrix acidic phosphoprotein 1 (*DMP1*)	Tumor suppressor gene from calmodulin kinase family with broad function in cell signaling;[52] extracellular matrix protein important for biomineralization;[56, 57] expressed in cancer with predilection for bone metastases (eg. human lung cancer)[58]	3.01 (0.032)

### Bone marrow differential gene expression

Overall 12 of 17 differentially expressed genes (relative to unaffected animals) in SAO^+^ BM were found to be associated with skeletal formation (Table 1). Sixteen of the transcripts were increased in SAO, with decreased expression only found in hydroxysteroid (17-beta) dehydrogenase 6, (*HSD17B6*). Among the genes with increased transcripts, *Equus caballus* solute carrier family 9, subfamily A2 (SLC9A2) expression is linked to osteoclast differentiation and survival.[27] Transient receptor potential cation channel, subfamily V, member 4 is a marker of osteoclast differentiation (*TRPV4*).[36] Osteomodulin (*OMD*) [28] and distal-less homeobox 3 (*DLX3*)[30] gene products are transcriptional indicators of osteoclast activity and differentiation, respectively. Death-associated protein kinase 2 (*DAPK2*),[41, 52] was the only genetic footprint of response to pro-inflammatory stimulus in BM of SAO^+^ horses. Five of the differentially expressed genes associated with the developing skeleton include *DLX3*,[59] Sp7 transcription factor (*SP7*),[40, 41] collagen type XXIV, alpha 1 (*COL24A1*),[37] collagen type XI, alpha 2 (*COLXIA2*),[47] and chondroadherin (*CHAD*). [48] Increased transcription of bone formation markers included integrin binding sialoprotein (*BSP*),[60, 61] osteocalcin (*OC*),[62] and sphingomyelin phosphodiesterase 3 (*SMPD3*).[45, 46]

### Tracheobronchial lymph node differential gene expression

Three out of 36 differentially expressed genes in the tLNs from SAO^+^ horses were included for their functional relevance to the silicosis induced inflammatory process (Table 2). Six of the 36 genes were uncharacterized proteins. The other 27 genes were broadly associated with immune functions and demonstrated a nonspecific pattern of differential expression (S3 Table).

### Gene ontology (GO) enrichment analysis of bone marrow and tracheobronchial lymph node transcriptomes

**Fig 1:**
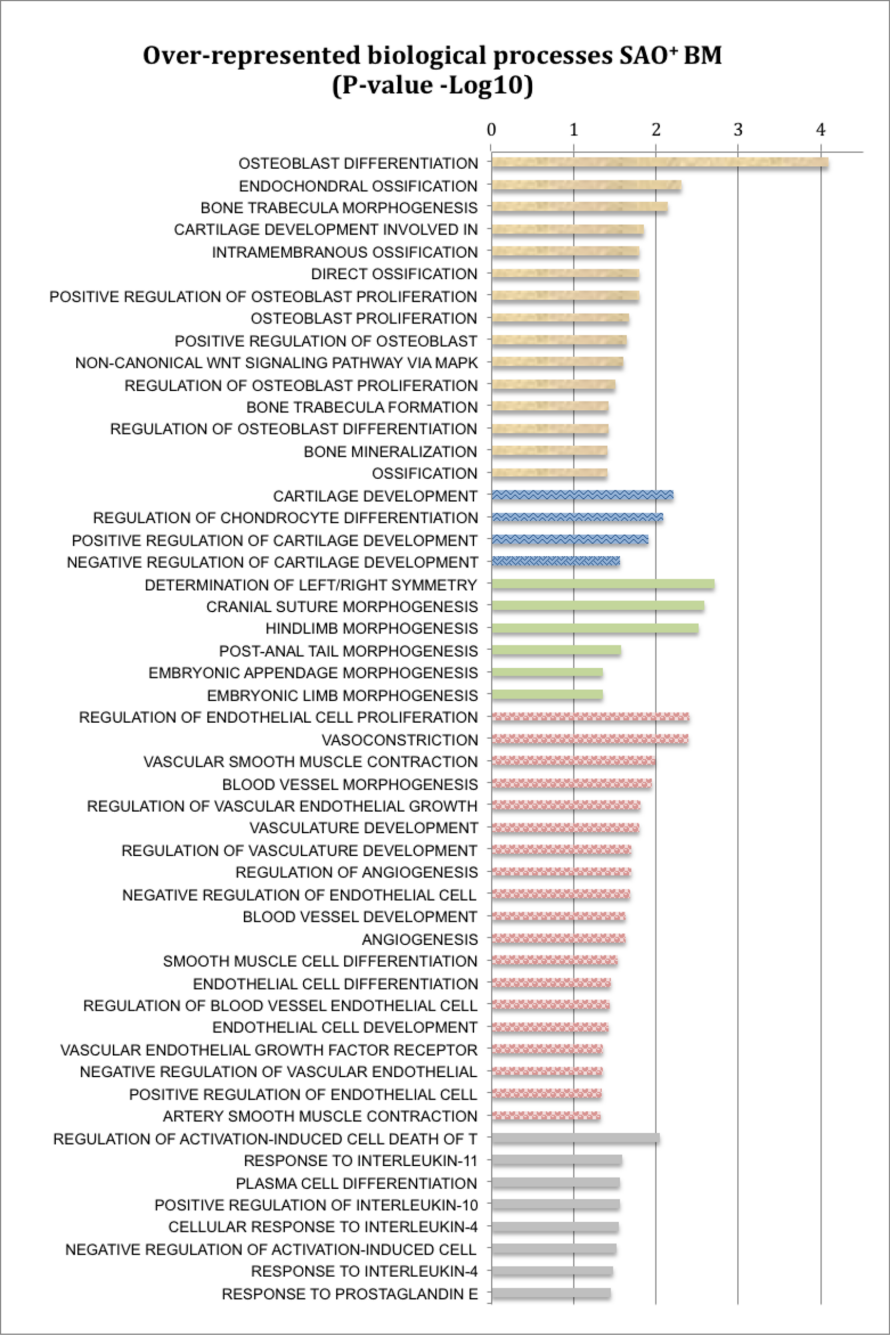
The selected, significantly over-represented biological processes in SAO^+^ BM sample (based on raw p value <0.05). The biological processes are listed in arbitrary categories associated with formation of bone (tan), cartilage (blue), embryonic skeleton (green), vessels (pink), and immune system responses (gray). Bars represent negative Log 10 conversion of the p value to illustrate the confidence in their over-representation in SAO^+^ BM. S4 Table includes the entire list of all the significant biological functions for BM GO enrichment analysis.

GO analysis probed the entire transcriptome of SAO^+^ and unaffected tissues against the structured dataset of biological functions to capture the significant differences in gene signaling and interactions relative to biological processes. The data is presented in raw p-value for the Kolmogorov-Smirnov test for the over-represented GO terms (S4 and S5 Tables). (Raw p-values are reported because there is no multiple testing correction method for hierarchically arranged GO terms). Differences in biological functions were identified in BM and tLN tissues. The order (P-value) revealed the statistical significance in the enriched biological function (S4 and S5 Tables). In BM transcriptome, 326 enriched biologic functions associated with SAO had P values <0.05 (S4 Table). The smallest p-value associated with enriched biological function was osteoblast differentiation (p = 8.20E-05) with other top functions related to skeletal and vascular systems development (Fig 1 and S4 Table). In the affected tLN, 448 enriched biological functions (P value <0.05) were broadly divided into immunological activation and few indicators of infection (S5 Table, S4 Fig).

### Panther analysis: PANTHER enrichment test

Increased signaling through the integrin (P00034, p = 5.91E-03), PDGF (P00047, p = 3.44E-02) and cadherin (P00012, p = 6.58E-03) pathways were found in the SAO^+^ BM samples compared to unaffected samples. Down-regulation of T cell activation (P00053, p = 1.51E-03), increased coagulation (P00011, p = 7.87E-03), and decreased signaling through PDGF (P00047, p = 7.92E-03) were over-represented pathways in affected tLN samples.

### Morphological link to transcriptome patterns informed through DGE and GO

BM and tLN sampled from the same site as tissues harvested for RNA were evaluated microscopically to assess the biological relevance of the transcriptome data.

#### Bone marrow

**Fig 2:**
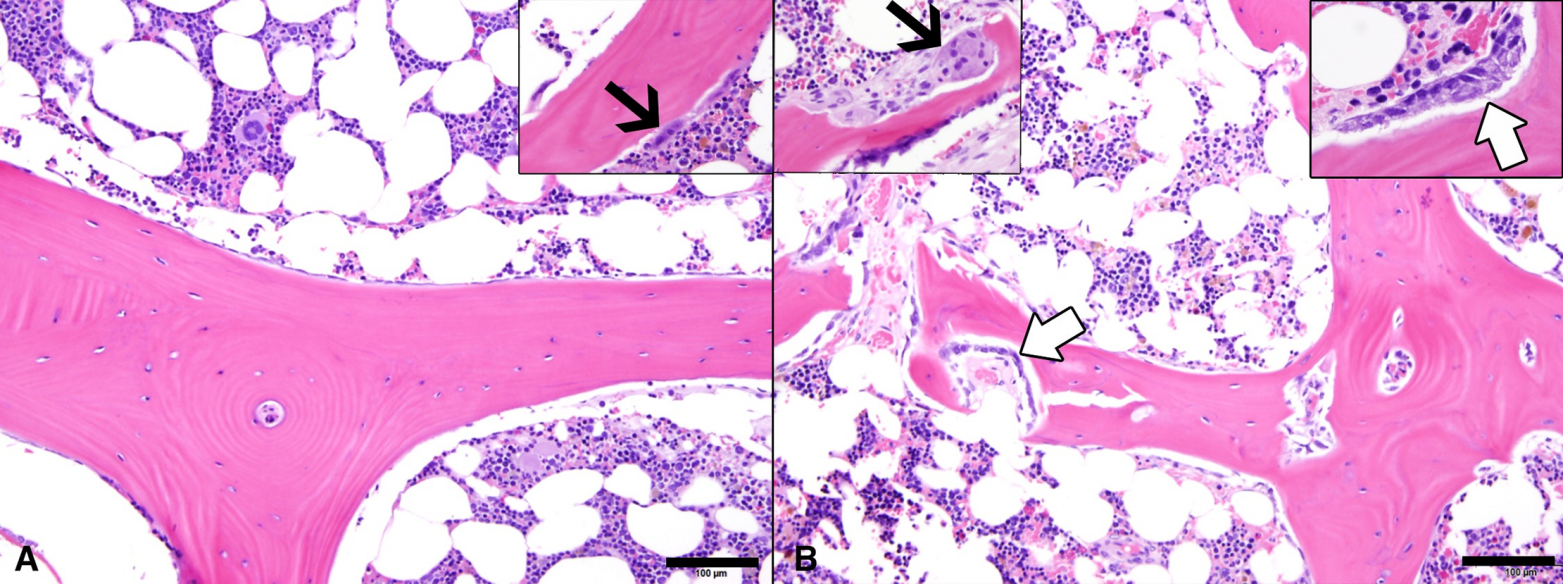
**Representative photomicrographs of decalcified unaffected (A) and SAO**^**+**^ **(B) BM sections.** Inter-trabecular BM stroma contained similarly mixed cell populations. A thin layer of bone lining cells cover the trabecular bone surfaces in the control BM sample (A). Rows of osteoblasts (white arrows) line some of the bone surfaces in SAO^+^ (B) and fewer surfaces in control (not shown) BM specimens. Rare osteoclasts (black arrows) are noted in the specimens but the osteoclasts and associated resorption bays appear larger in SAO^+^ BM. [H&E stain; Scale bars 100 μm].

Bone formation, resorption, and total perimeters were quantified in SAO^+^ and unaffected BM samples. Mean (± SD) ratio of bone forming surface to total bone surface perimeter was greater (p<0.001) in SAO^+^ horses (0.31 ± 0.14) than unaffected horses (0.05 ± 0.04). Osteoblast hypertrophy, captured as apical to basilar height, was significantly greater (p = 0.002) in SAO^+^ horses (11.6 ± 2.2 μm) than unaffected horses (5.4 ± 3.8 μm). Mean ratio of bone resorption surface to total bone surface perimeter was greater (p = 0.016) in SAO^+^ horses (0.15 ± 0.12) than unaffected horses (0.03 ± 0.04). However, few osteoclasts were present in sections (ranging from 0 to 6 per image area) compared to the other cell populations and numbers per total bone perimeter were not statistically different (p = 0.401) between affected and unaffected horses. However, osteoclasts were subjectively more ubiquitous in SAO^+^ horses with greater cell volume and nuclei numbers (Fig 2). Cartilaginous tissue was not identified in the rib sections.

#### Tracheobronchial lymph nodes

**Fig 3:**
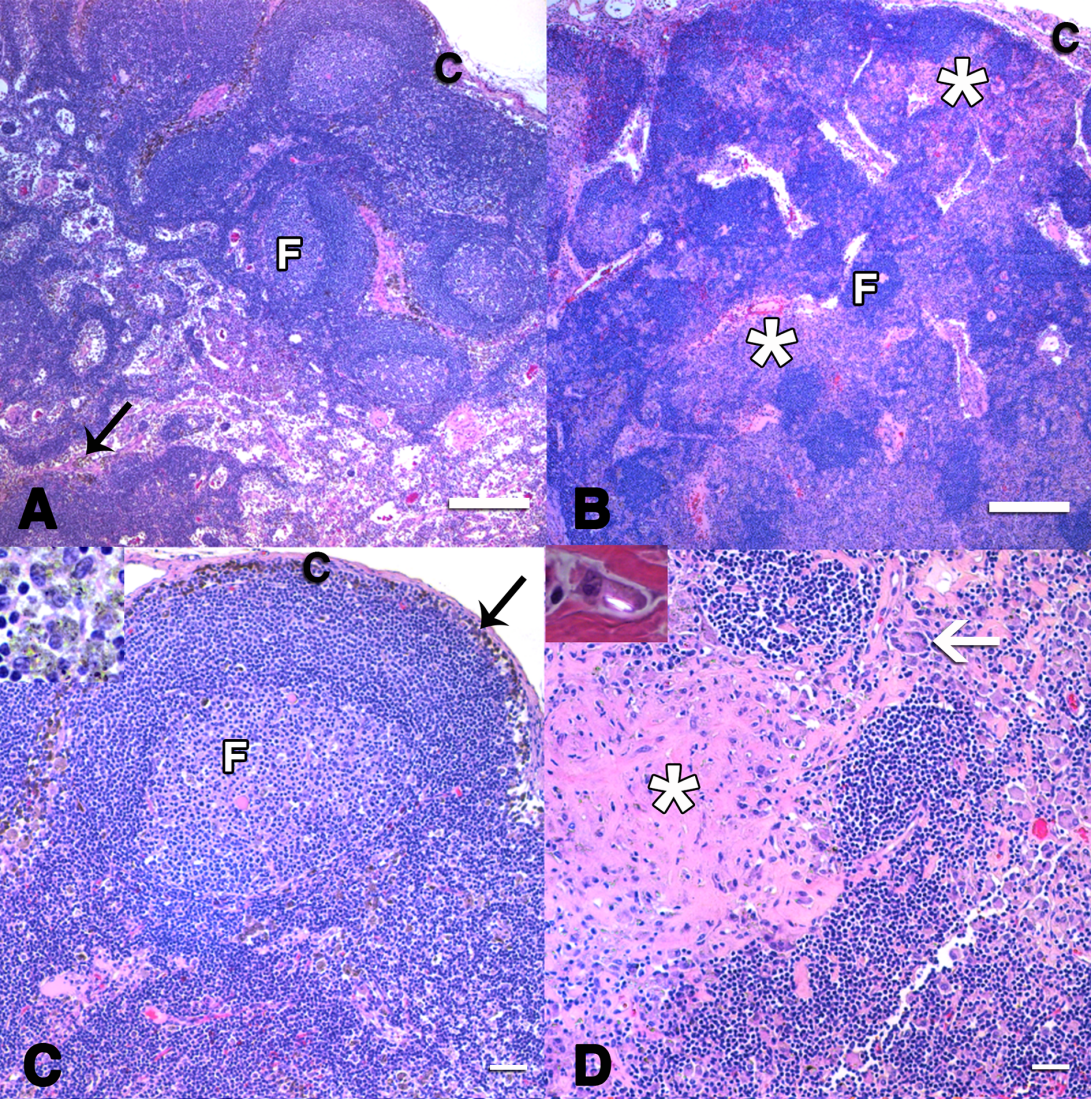
**Photomicrographs of unaffected (A, C) and SAO**^**+**^ **tLNs (B, D).** The affected tLN had regions of pallor in the cortex and medullary cords (asterisks) subjacent to atrophied lymphoid follicles (“F”, B). Abundant mature and smudged collagen deposits (fibrosis) co-localized with numerous macrophages, giant cells (arrow, D) and refractile, sharp crystalline particles (inset—high magnification of macrophage under polarized light, D). Granular, pigmented crystals (black arrows and inset, A, C) consistent with incidental anthracosilicosis were noted in the macrophages of both unaffected and affected tLNs (not shown in B, D) without inflammation, necrosis and fibrosis characteristic of reaction to cytotoxic silicate crystals. Infectious organisms were not found in any lymph nodes. (c–lymph node capsule) [H&E stain; Scale bars: (A,B) 500μm; (C,D) 50μm].

The lymphoid follicles in SAO^+^ horses were atrophied compared to the well-defined follicular architecture present in unaffected tLN samples (Fig 3). Mild to moderate granulomatous inflammation and fibrosis obscured large regions of the tLN medullary cords and cortex in the SAO^+^ group (Fig 3). Variable numbers of thin, refractile crystals (<2 μm long) were identified within regions of inflammation and fibrosis under polarized light in tLNs only from affected horses. These crystals were non-pigmented and indistinct under transmitted white light. Different refractile, brown pigmented crystals were observed in 10–30% of the sinusoidal macrophages of all tLNs. Necrosis, fibrosis, multinucleated macrophages, and inflammation were not associated with these crystals in tissues.

### Metatranscriptomic analysis

To determine if the presence of infectious agents was associated with the differential transcriptome data from SAO^+^ and unaffected horses, metatranscriptomic analysis was conducted. In all samples, reads assigned to *Equus caballus* comprised greater than 99.8% of all reads or read pairs. The most abundant, non-equine species present in affected and control BM and tLN tissues included *Choristoneura occidentalis granulovirus* (an arthropod virus, <0.09% of reads) and *Alteromonas mediterranea* (a marine bacterium, <0.02% of reads). One SAO^+^ BM sample had *Mycobacterium tuberculosis* as the second most abundant species (0.00017% of reads) without microscopic evidence of granulomatous lesions.

### Viral discovery

Metagenomic analysis was conducted on selected tissues from 3 SAO^+^ horses to determine if viral genetic material could be detected. No viral RNA, despite the findings of *granulovirus* in the metatranscriptomic analysis, was identified in pooled tissues (BM, tLN, blood, and lung).

## Discussion

The cardinal lesions of SAO in tLN and bone marrow-rich skeletal sites were present in all affected horses, supporting circumstantial evidence for their interconnected role in the pathobiology of SAO.[1] The molecular signature within the microenvironment of affected pulmonary and skeletal tissues was explored to enhance the understanding of SAO disease pathogenesis. RNA-seq was used in the investigation of BM and tLN transcriptomes. To compare the pattern of molecular signatures with the biological processes within BM and tLN tissues, transcriptome results were assessed in light of the histologic features in the sites used for molecular analysis. Several transcripts associated with bone formation (e.g., increased osteoblast-associated activity) and a lesser number for bone resorption were overrepresented in SAO^+^ BM transcriptome relative to unaffected animals. DGE and GO enrichment analyses demonstrated pro-inflammatory activity in SAO^+^ tLNs, but inherent tissue heterogeneity may have diluted a more distinct transcriptome pattern. There was no evidence of infectious co-factors in SAO etiology. This study provides a novel viewpoint of a dominant molecular signature of bone formation (increased osteoblastic activity) in a condition that has been morphologically defined by osteoclastic hyperactivity and clinically significant osteoporosis.[1] This data introduced an unexpected conundrum.

Although unexpected, the paucity of transcripts associated with osteoclast differentiation in SAO^+^ BM tissue suggests a number of pathogenic possibilities. Lack of anticipated transcriptome pattern associated with increased signaling through pro-osteoclastogenic RANK may reflect the low frequency of these transcripts within the complex cellular milieu of BM. Alternatively, the lack of evidence for a dominant osteoclastogenic signal may reflect the brief temporal nature of such a signal. Regardless, within lesions characterized by abundant bone removal, histologic evidence of overt osteoblastic activity is concurrently present. By design, tissue RNA samples were obtained from grossly normal bone, anatomically distant from osteoporotic lesions.

DGE and GO enrichment analyses of the SAO^+^ BM transcriptome revealed predilection for skeletal formation transcription. This finding was supported by morphologically increased osteoblast activity in affected BM samples compared to quiescent bone surfaces of unaffected tissues. Osterix (*Sp7*) is one of the differentially increased transcripts indicative of enhanced osteoblast differentiation.[41] Expression of osteoblast activity markers downstream of *Sp7* like *IBSP*, *BGLAP* and *COL24A1* provided further transcriptional evidence of increased osteoblast differentiation in SAO^+^ BM.[43, 63, 64]

Marked osteoblastic activity coupled with hyperactive osteoclasts is characteristic of SAO lesions and other bone diseases like fibrous osteodystrophy. However, overrepresented activation of osteoblasts away from regions of intense osteolysis was not anticipated. Activation of osteoblasts as a systemic reparative or compensatory mechanism despite minimal osteoclastic activity within tested sample is a potential explanation. The results could be considered in the context of increased mechanical loading distributed along weakened bone and increased differentiation of BM stromal cells toward osteo-chondral lineage.[65] The induction of osteoblast differentiation by pro-inflammatory cytokines like Il-1β was also a new consideration.[66] Trabecular osteocytes interact with BM stroma and may add to the pool of differentially expressed genes like *DLX3* and *CDH15* shown to have a higher transcription level than osteoblasts in one study.[32]

Mechanisms involved in fetal bone formation are rekindled during endochondral ossification in skeletal fracture repair and potentially activated in SAO^+^ BM. Increased transcripts in our data are downstream of the master regulators of skeletal formation like bone morphogenetic protein (BMP) (reviewed by V.S. Salazar) [67] and runt related transcription factor 2 (Runx2) [68], (reviewed by T. Komori).[69] Transcriptional pattern of osteo-chondroprogenitor cell differentiation is reflected in the DGE signature and enriched biological functions associated with chondrocyte development and endochondral ossification despite histological absence of cartilage in BM samples. For example, genes such as *COLXIA2*, *SMPD3*, *TRPV4*, *CHAD*, *and ANO5* are associated with cartilage tissue functions [45, 47] although cartilage and endochondral ossification have not been reported in SAO osteolytic lesions. Skeletal tissue-stereotypic genetic regulations of repair and embryonic pathways of the skeletal formation likely overlap regardless of the mechanism of injury. The roles for the genes associated with endochondral ossification and skeletal morphogenesis might be expanded as transcription patterns within SAO bone lesions and appendicular skeletal sites rarely affected by osteoporosis are elucidated in the future.

Increased osteoclast differentiation and activity in SAO^+^ BM was speculatively linked to the increased transcription of *SLC9A2*, *DLX3*, *TRPV4* and *OMD*.[27, 30, 36] The latter gene is up regulated in an osteoblast response to osteoclastic activity.[28] Results from GO analysis did not demonstrate biological functions associated with osteoclasts. The sensitivity of the transcriptome analysis might not be sufficient to detect differential expression of osteoclasts outnumbered in BM samples by other cell populations. Alternatively, osteoclast transcriptome may be sample-dependent and thus indicative of multifocal rather than generalized nature of the pro-osteoclastic differentiation and osteolysis in SAO.

Our results suggest a multifocal rather than systemic activation of an osteolytic trigger in SAO. The multifocal distribution of osteolysis has been noted in post mortem examination and on nuclear scintigraphic examination of SAO^+^ horses, but the potential significance has not been realized.[1, 3] The putative differential transcriptome expression pattern may only be captured within osteolytic lesions and would be sample site dependent. Additionally, the histogenic nature of the cytologically atypical, multinucleated, osteolytic cells in SAO may need to be defined beyond microscopic phenotype together with the histogenesis of their precursors. Nevertheless, our results bring attention to osteoblasts and their multidimensional role in bone tissue formation, regulation of their microenvironment, and implication in pathogenesis of bone fragility diseases.

The role of the only down-regulated transcript, *HSD17B6*, in SAO^+^ BM is unclear because function of the equine isoenzyme is not defined in the literature. *HSD17B6* might have an important role in bone as some isoenzymes catalyze the interconversion of estrone (E1) to “osteoprotective” estradiol E2).[51] In human and mice prostate tissues, *HSD17B6* has been identified as a mediator of the conversion of androgen substrates into inactive forms.[70] Depending on the substrate and the product of the hydroxysteroid dehydrogenase, depletion of *HSD17B6* in SAO^+^ horses could be a response to accelerated bone loss, or part of the disease pathobiology resulting in excessive osteolysis.

Compared to GO and DGE analysis, PANTHER pathway evaluation of the BM transcriptome did not explicitly demonstrate osteoblast-associated functions. Pathway analysis showed increased signaling through the integrin and cadherin pathways broadly used by cells in the BM tissue.

Both DGE and GO enrichment analyses showed evidence for nonspecific inflammation in the tLNs of SAO^+^ horses. DGE analysis did not reveal a transcription pattern to explain the association between the skeleton and lung conditions in SAO. The 30 functionally defined differentially expressed genes within the SAO^+^ tLNs showed variably regulated transcripts broadly related to immune system functions. Theoretically, connections between silicosis and osteoporosis could be made with some transcripts. For example, an increase in *DMP1* transcription echoes BM tropism of an osteolytic trigger in SAO because it was up-regulated in bone tropic metastatic lung cancers.[58] Physiologic expression of *DMP1* in non-mineralized tissues may indicate a wider purpose for extra-skeletal regulation of mineralization.[71] *PTX3* up-regulation may have some connection with silicosis as a promoter in TNF-α and IL-1β production through binding NF-κB elements, as demonstrated *in vitro*.[54] Increased production of IL-1 and TNF-*α* has been implicated in the pathobiology of silicosis.[72, 73] Interestingly, *PTX3* is increased in cultured human bronchial epithelial cells exposed to cytotoxic silicates.[74] Inherent cell population variability within affected and unaffected tLN tissues may have distorted the signal pattern of the silicosis and diluted the significant differentially expressed genes. Heterogeneity included regions where more than 40% of affected tLN had been replaced by fibrosis, necrosis, and granulomatous inflammation in response to intralesional refractile crystals (presumed cristobalite).

Modification of the tissue target, sample collection, and RNA preparation protocols, as well as identification of SAO clinical markers would curtail some of the limitations for the prospective investigation of this condition. Because necropsy and histopathology remain the gold standard diagnostic tools to confirm SAO, only horses in terminal condition were recruited for the study. Concerns over transportation of severely affected SAO^+^ horses with advanced osteoporosis resulted in challenging barriers for patient recruitment. Isolation of the specific cell targets from BM and blood of live animals, such as osteoblasts or osteoclasts using laser dissection, and monocytes with a cell sorting technique may prove useful for future studies. The implication of the results from the metatranscriptomic analysis in our study was unclear since the low frequency transcripts of an arthropod virus and marine bacteria were present in all the samples. Misclassification of the non-equine transcripts was considered as a possibility. The finding of low frequency transcripts of *Mycobacterium tuberculosis* in one SAO^+^ BM sample was not supported by clinical and pathological examination. Elimination of the poly A RNA selection step would potentially resolve limitations of the metatranscriptomic analysis in including infectious agents that do not transcribe their genetic material in this RNA form.

In conclusion, the data revealed an SAO associated transcriptome profile in BM tissue skewed toward skeletal formation which is also supported by microscopic findings (at bone sites distinct from lesions of osteolysis). The hypothesized molecular signal of a pathogenic link connecting the pulmonary and skeletal lesions was not elucidated by our results, and increased transcripts associated with osteoclast differentiation and activation were relatively fewer than osteoblast related transcripts. A clear transcriptome pattern was not identified between affected and unaffected animals due to the cellular heterogeneity in the tLN samples. Specific cell targets from the BM and tLN tissues would address the potential dilution of the disease-specific transcriptome pattern. Transcriptome profile of osteoclasts, osteoclast precursors or macrophages from osteolytic SAO lesions may provide better understanding of osteoclast dysregulation. The effect of ongoing pulmonary inflammation and silicosis on osteoblasts and regulation of bone formation warrants investigation in light of our findings. This is the first study that uses RNA-seq technology in investigation of disease mechanism in the equine co-morbid condition though RNA-seq has been used in research of other equine diseases.[75–79] SAO may provide an opportunity for a unique understanding of mechanisms in an environmentally induced condition with pulmonary/tLN and skeletal comorbidity.

## Supporting information

S1 Fig BM and tLN MDS plot. The sequenced samples transcript patterns cluster based on tissue in this MDS plot that simultaneously evaluated BM and tLN transcriptomes together.
(TIF)

S2 Fig BM MDS plot. BM MDS plot demonstrates clustering of cases (red) based on bone phenotype. The group with mild osteoporosis (encircled in black) co-localized with control cases.
(TIF)

S3 Fig tLN MDS plot. tLN MDS plot demonstrates clustering of control cases and wider dispersal of the cases transcriptome profiles.
(TIF)

S4 Fig tLN GO enrichment chart. The most enriched biological processes related infectious agents like virus and bacterial elements in the SAO^+^ tLN with raw p-value <0.05 are included in the bar chart. Bars represent -Log _10_ converted p values. S5 Table includes the entire list of all the significant biological functions for tLN GO enrichment analysis.
(TIF)

S5 Fig Sex and age do not have effect on distribution of BM transcriptome in the MDS plot. (TIF)

S6 Fig Sex and age do not have effect on distribution of tLN transcriptome in the MDS plot. (TIF)

S1 Table Horses information. Animal signalment and quality of RNA extracted from BM (BM RIN) and tLN (LN RIN) for SAO^+^ and Control horses.
(DOCX)

S2 Table Differentially expressed genes in SAO+ BM tissue. (XLSX)

S3 Table Differentially expressed genes in SAO+ tLN tissue. (XLSX)

S4 Table Enriched biological functions in the SAO+ BM. (XLSX)

S5 Table Enriched biological functions in the SAO+ tLN. (XLSX)

S1 Protocol Metagenomic analysis, viral discovery supplemental methodology description. (DOCX)

## Abbreviations

BM: bone marrow
DGE: differential gene expression
GO: Gene ontology
M: mild SAO^+^ phenotype
MDS: Multidimensional scaling plot
RIN: RNA (ribonucleic acid) integrity score
RNA-seq: RNA sequencing
S: severe SAO^+^ phenotype
SAO: Silicate Associated Osteoporosis
SAO^+^: tissues and samples from horses positive for SAO phenotype
SAO^-^: tissues and samples from horses negative for SAO phenotype, and without exposure to the SAO endemic regions
tLN: tracheobronchial lymph node
