# Supplementary figures and images for: Bone formation transcripts dominate the differential gene expression profile in an equine osteoporotic condition associated with pulmonary silicosis
Source: PLoS One. 2018 Jun 1;13(6):e0197459. doi: 10.1371/journal.pone.0197459 (PMC5983561; doi:10.1371/journal.pone.0197459)

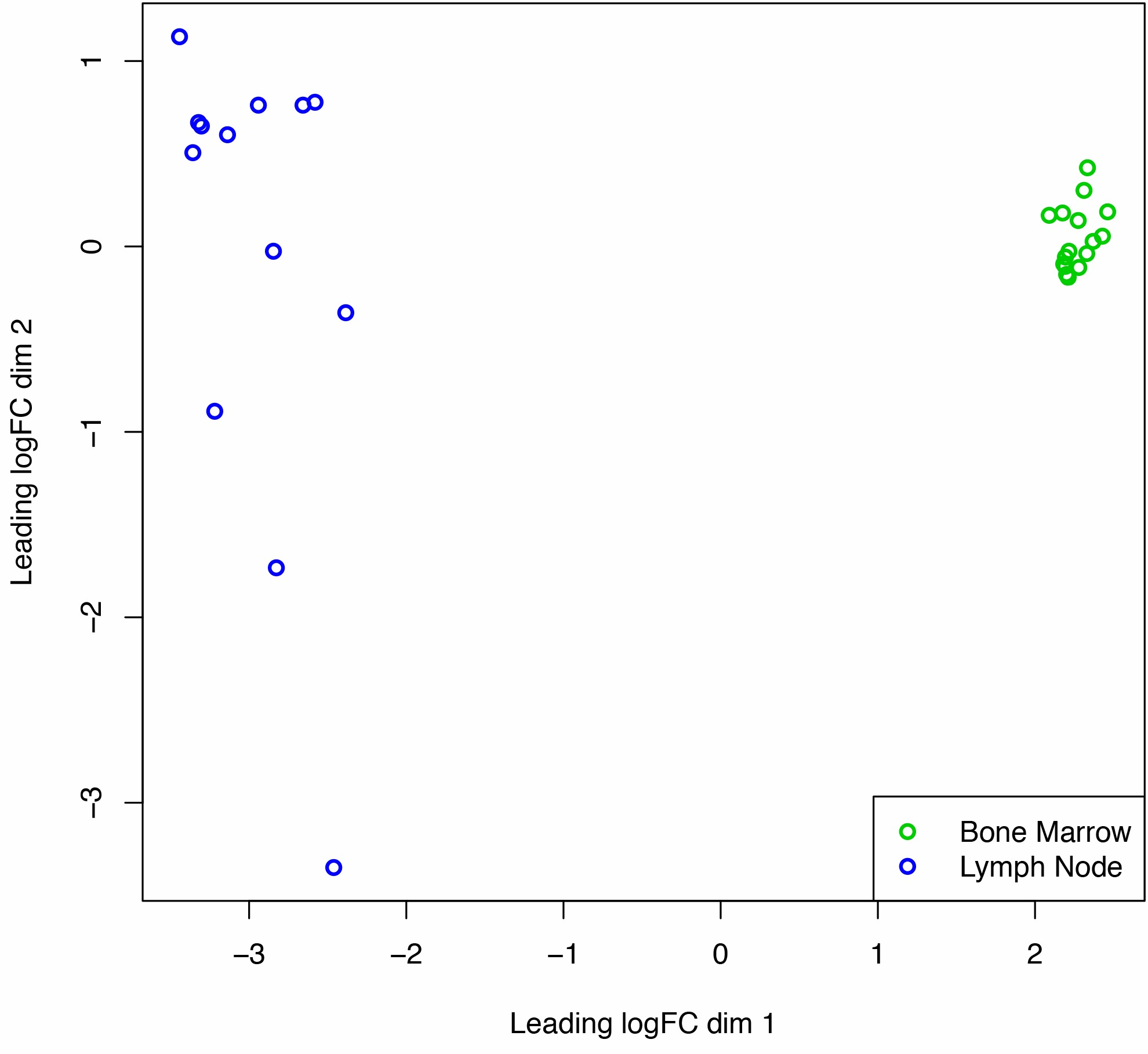

Supplement: S1 Fig — The sequenced samples transcript patterns cluster based on tissue in this MDS plot that simultaneously evaluated BM and tLN transcriptomes together. (TIF) [file pone.0197459.s001.tif]

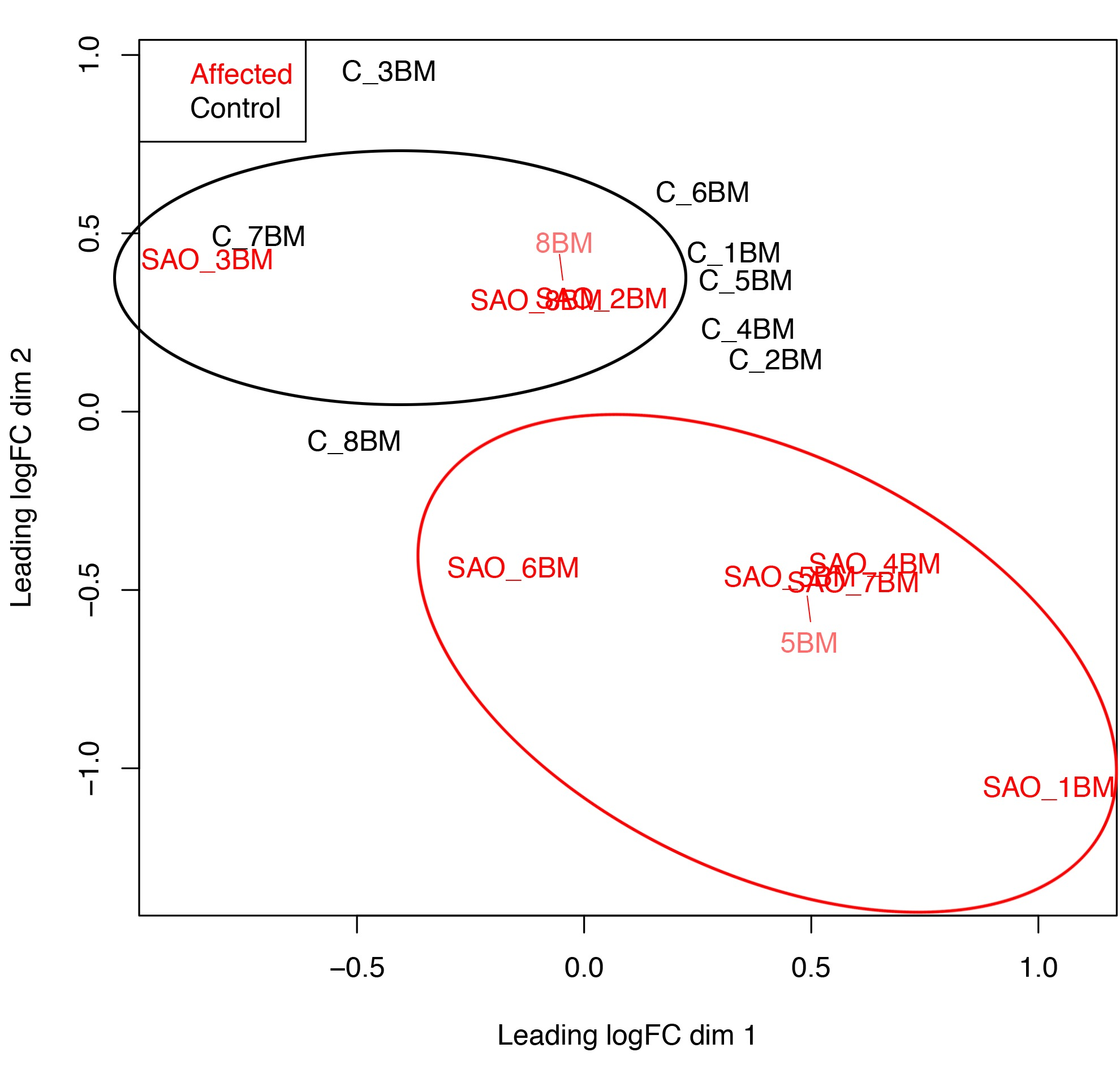

Supplement: S2 Fig — BM MDS plot demonstrates clustering of cases (red) based on bone phenotype. The group with mild osteoporosis (encircled in black) co-localized with control cases. (TIF) [file pone.0197459.s002.tif]

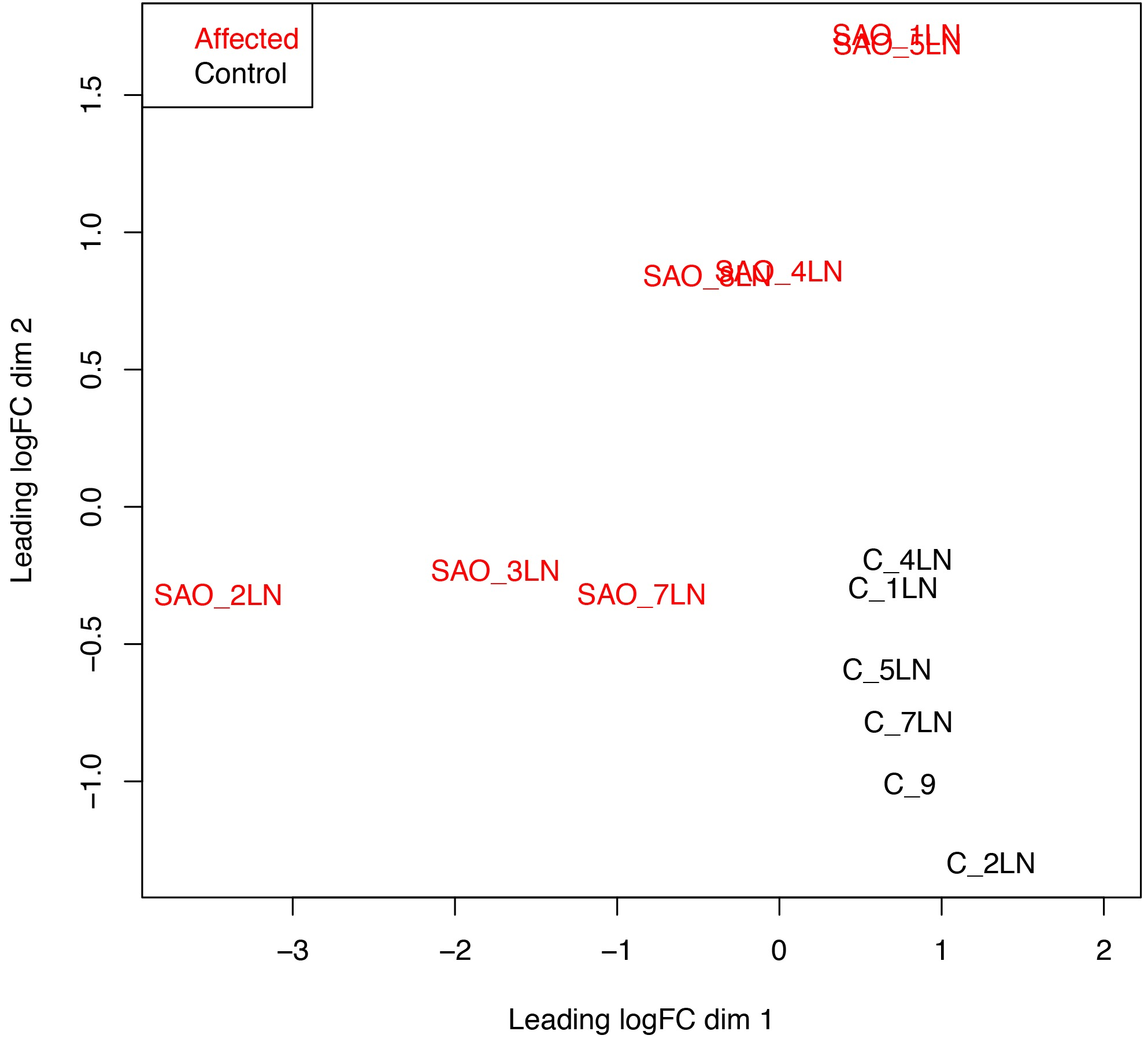

Supplement: S3 Fig — tLN MDS plot demonstrates clustering of control cases and wider dispersal of the cases transcriptome profiles. (TIF) [file pone.0197459.s003.tif]

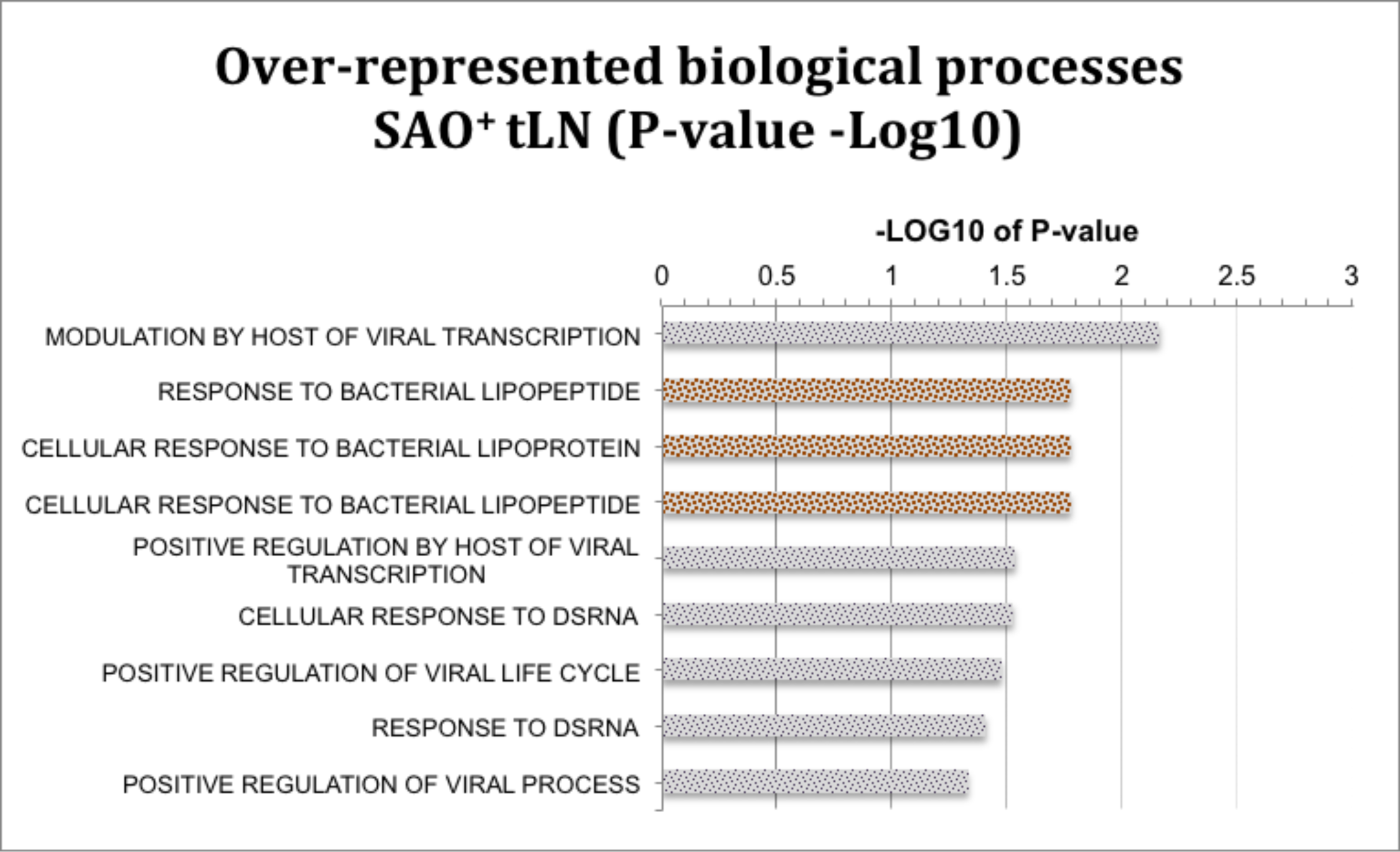

Supplement: S4 Fig — The most enriched biological processes related infectious agents like virus and bacterial elements in the SAO+ tLN with raw p-value <0.05 are included in the bar chart. Bars represent -Log 10 converted p values. S5 Table includes the entire list of all the significant biological functions for tLN GO enrichment analysis. (TIF) [file pone.0197459.s004.tif]

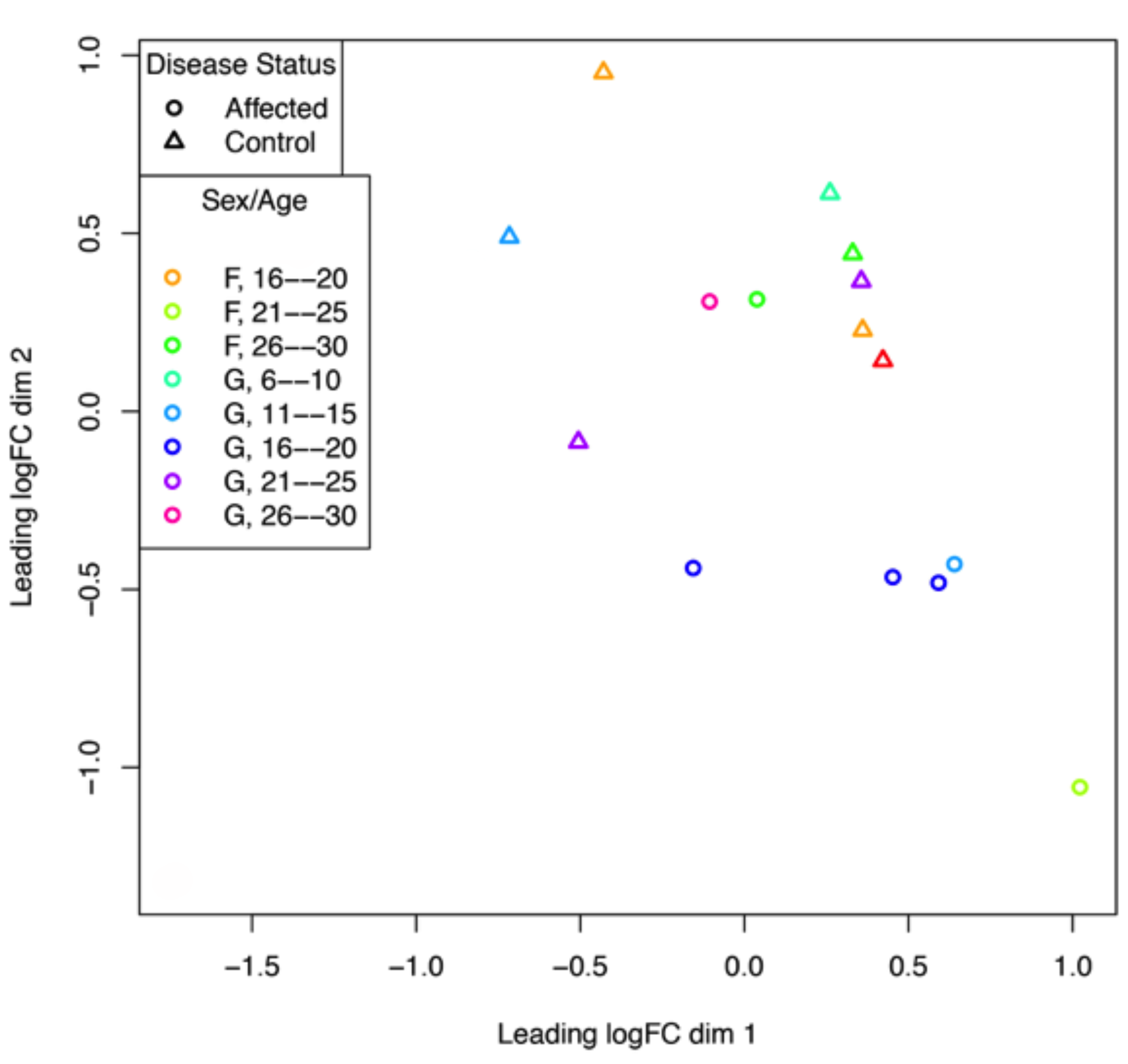

Supplement: S5 Fig — (TIF) [file pone.0197459.s005.tif]

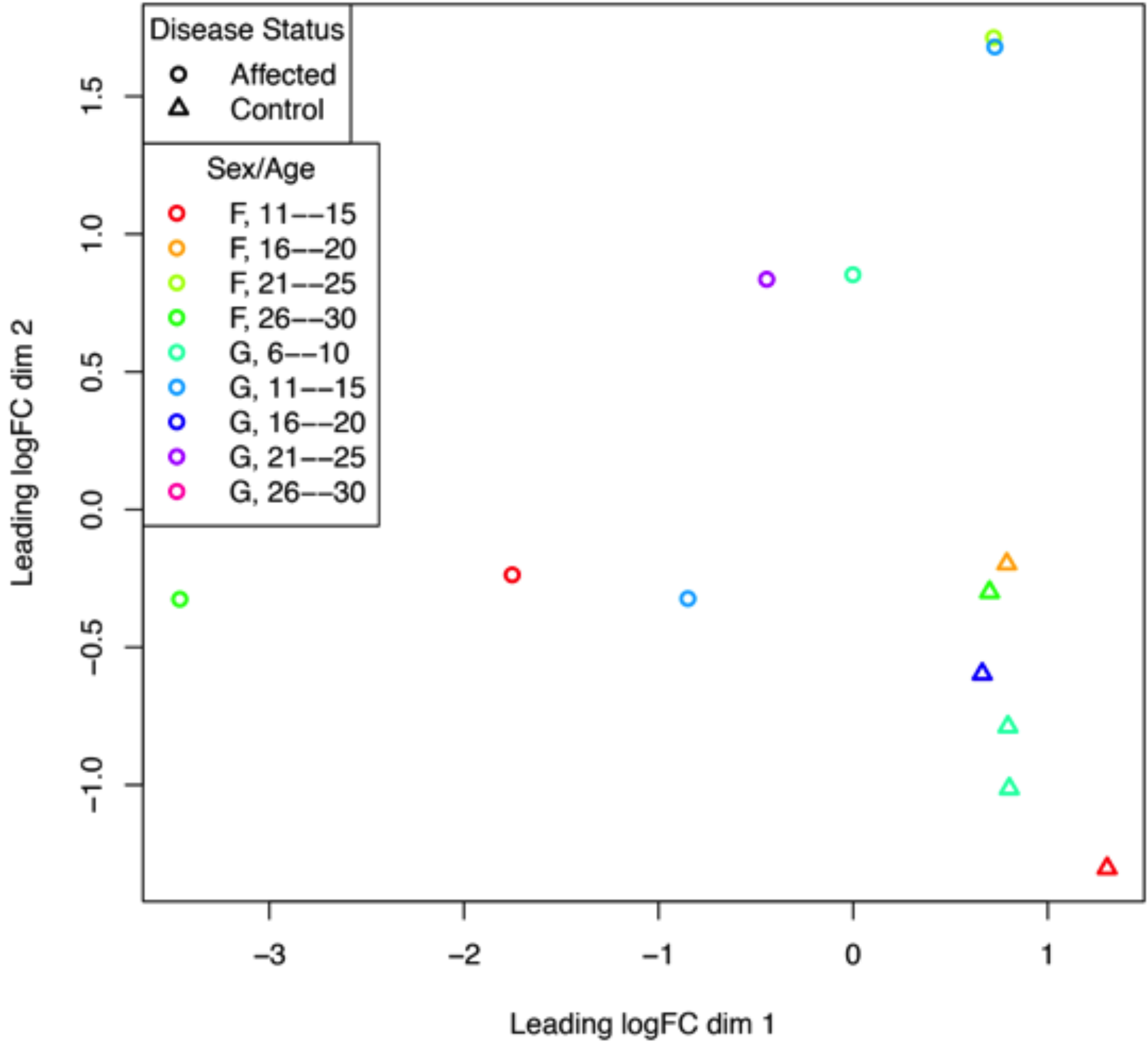

Supplement: S6 Fig — (TIF) [file pone.0197459.s006.tif]
