# Supplementary material for: Bone formation transcripts dominate the differential gene expression profile in an equine osteoporotic condition associated with pulmonary silicosis
Source: PLoS One. 2018 Jun 1;13(6):e0197459. doi: 10.1371/journal.pone.0197459 (PMC5983561; doi:10.1371/journal.pone.0197459)
Supplement: S1 Table — Animal signalment and quality of RNA extracted from BM (BM RIN) and tLN (LN RIN) for SAO+ and Control horses. (DOCX) [file pone.0197459.s007.docx]

| **Table S1.** Animal signalment and quality of RNA extracted from BM (BM RIN) and tLN (LN RIN) for SAO^+^ and Control horses. | | | | | | | | | | | |
| --- | --- | --- | --- | --- | --- | --- | --- | --- | --- | --- | --- |
|  | **SAO^+^ Case** | **Breed** | **Age(yr)** | **BM**  **RIN** | **LN**  **RIN** |  | **Controls** | **Breed** | **Age (yr)** | **BM**  **RIN** | **LN RIN** |
| Females | | | | | | | | | | | |
| 1 | **SAO1 (S)*** | Appal† | 24 | 8.9 | 8.9 | 1 | C1 | Hanov - Nǂ | 26 | 9 | 9.3 |
| 2 | SAO2 (M) | QH | 30 | 8.3 | 8.7 | 2 | C2 | TB - MS | 11 | 8.6 | 9.3 |
| 3 | SAO3 (M) | TB | 12 | 8.3 | 8.9 | 3 | C3 | Paint - B | 18 | 9 | NA** |
|  | | | | | | 4 | C4 | QH - MS | 17 | 8.5 | 9.4 |
| Males | | | | | | | | | | | |
| 1 | **SAO4 (S)** | Holst | 13 | 9.5 | 9.3 | 1 | C5 | QH - MS | 25 | 8.9 | 9.4 |
| 2 | **SAO5 (S)** | QH/X | 18 | 8.1 | 8.9 | 2 | C6 | American  SB/Arab - MS | 8 | 9.6 | NA |
| 3 | **SAO6 (S)** | QH | 20 | 9.1 | NA | 3 | C7 | Mustang/X - MS | 15 | 7.5 | 9.4 |
| 4 | **SAO7 (S)** | Paint | 20 | 8.3 | 8.2 | 4 | C8 | Morgan/Arab - F | 25 | 8.3 | NA |
| 5 | SAO8 (M) | Arab | 30 | 9.3 | 8.9 | 5 | C9 | Holst - N | 13 | NA | 9 |

* Osteoporosis grade - (S) severe, (M) mild

† Breeds: Holst – Holsteiner; Hanov – Hanoverian; Appal – Appaloosa; Arab – Arabian; QH - Quarter horse; TB – Thoroughbred; SB – Saddlebred; X - Mixed breed

ǂ Reasons for euthanasia of control horses are abbreviated as follows: N – neurological; MS – musculoskeletal (lameness and chronic pain); B – Behavioral (dangerous to handle); F – fatal acute fracture.

** NA = RNA was not obtained or sequenced due to poor tissue quality.
