# Supplementary material for: Bone formation transcripts dominate the differential gene expression profile in an equine osteoporotic condition associated with pulmonary silicosis
Source: PLoS One. 2018 Jun 1;13(6):e0197459. doi: 10.1371/journal.pone.0197459 (PMC5983561; doi:10.1371/journal.pone.0197459)
Supplement: S1 Protocol — (DOCX) [file pone.0197459.s012.docx]

**Supplemental methodology**

**Metagenomic analysis, Viral Discovery**

Bone marrow core, tracheobronchial lymph node from SAO^+^ cases SAO1, SAO7, SAO3, SAO9 (not used in transcriptome study)), as well as buffy coat (SAO7, SAO9) and lung (SAO1, SAO3) tissues were pooled. The tissues were ground, homogenized and re-suspended in 10 volumes of 1X PBS. Supernatant (400 µl) was collected after centrifugation (10 min, 15000 *g*), and filtered through a 0.45 µm filter (Millipore) to remove eukaryotic and bacterial cell-sized particles. The filtrates were treated with a mixture of DNases [Turbo DNase (Ambion), Baseline-ZERO (Epicentre), benzonase (Novagen)] and RNase (Fermentas) at 37 °C for 90 min to enrich for viral capsid-protected nucleic acids.[1] Nucleic acids were then extracted using magnetic beads of the MagMAX Viral RNA Isolation kit (Ambion) according to the manufacturer’s instructions. An Illumina MiSeq library was constructed using random RT-PCR followed by use of the Illumina Nextera kit and sequenced on an Ilumina MiSeq platform using 250 bases paired end. Sequence reads were de novo assembled using the Ensemble program and contigs and singletons then translated into hypothetical protein sequences, which were compared to all viral proteins in the NCBI virus RefSeq database using BLASTx.

1. Victoria JG, Kapoor A, Li L, Blinkova O, Slikas B, Wang C, et al. Metagenomic analyses of viruses in stool samples from children with acute flaccid paralysis. J Virol. 2009;83(9):4642-51. doi: 10.1128/JVI.02301-08. PubMed PMID: 19211756; PubMed Central PMCID: PMCPMC2668503.
